# RAGE inhibition alleviates lipopolysaccharides-induced lung injury via directly suppressing autophagic apoptosis of type II alveolar epithelial cells

**DOI:** 10.1186/s12931-023-02332-6

**Published:** 2023-01-24

**Authors:** Xi Xiong, Jiaying Dou, Jingyi Shi, Yuqian Ren, Chunxia Wang, Yucai Zhang, Yun Cui

**Affiliations:** 1 Department of Critical Care Medicine, Shanghai Children’s Hospital, School of Medicine, Shanghai Jiao Tong University, Shanghai, 200062 China; 2 Institute of Pediatric Infection, Immunity and Intensive Care Medicine, Shanghai Jiao Tong University, Shanghai, 200062 China; 3 Institute of Pediatric Critical Care, Shanghai Jiao Tong University, Shanghai, 200062 China; 4 Clinical Research Unit, Shanghai Children’s Hospital, Shanghai, 200062 China

**Keywords:** Receptor for advanced glycation end products, Type II alveolar epithelial cells, Autophagy, Acute lung injury

## Abstract

**Background:**

Advanced glycation end product receptor (RAGE) acts as a receptor of pro-inflammatory ligands and is highly expressed in alveolar epithelial cells (AECs). Autophagy in AECs has received much attention recently. However, the roles of autophagy and RAGE in the pathogenesis of acute lung injury remain unclear. Therefore, this study aimed to explore whether RAGE activation signals take part in the dysfunction of alveolar epithelial barrier through autophagic death.

**Methods:**

Acute lung injury animal models were established using C57BL/6 and *Ager* gene knockout (*Ager* ^−/−^ mice) mice in this study. A549 cells and primary type II alveolar epithelial (ATII) cells were treated with siRNA to reduce *Ager* gene expression. Autophagy was inhibited by 3-methyladenine (3-MA). Lung injury was assessed by histopathological examination. Cell viability was estimated by cell counting kit-8 (CCK-8) assay. The serum and bronchoalveolar lavage fluid (BALF) levels of interleukin (IL)-6, IL-8 and soluble RAGE (sRAGE) were evaluated by Enzyme-linked immunosorbent assay (ELISA). The involvement of RAGE signals, autophagy and apoptosis was assessed using western blots, immunohistochemistry, immunofluorescence, transmission electron microscopy and TUNEL test.

**Results:**

The expression of RAGE was promoted by lipopolysaccharide (LPS), which was associated with activation of autophagy both in mice lung tissues and A549 cells as well as primary ATII cells. sRAGE in BALF was positively correlated with IL-6 and IL-8 levels. Compared with the wild-type mice, inflammation and apoptosis in lung tissues were alleviated in *Ager*^−/−^ mice. Persistently activated autophagy contributed to cell apoptosis, whereas the inhibition of autophagy by 3-MA protected lungs from damage. In addition, *Ager* knockdown inhibited LPS-induced autophagy activation and attenuated lung injury. In vitro, knockdown of RAGE significantly suppressed the activation of LPS-induced autophagy and apoptosis of A549 and primary ATII cells. Furthermore, RAGE activated the downstream STAT3 signaling pathway.

**Conclusion:**

RAGE plays an essential role in the pathogenesis of ATII cells injury. Our results suggested that RAGE inhibition alleviated LPS-induced lung injury by directly suppressing autophagic apoptosis of alveolar epithelial cells.

**Supplementary Information:**

The online version contains supplementary material available at 10.1186/s12931-023-02332-6.

## Background

Acute lung injury (ALI) and acute respiratory distress syndrome (ARDS) secondary to primary pulmonary infection contribute significantly to morbidity and mortality [1]. Alveolar epithelial cells (AECs) injury is a critical hallmark of ALI/ARDS, which cover more than 95% of the internal surface area of the lung [2]. Exploring the potential mechanisms of lung epithelial cell injury can provide new insight into ARDS.

Gram-negative bacterial infection is one of the most important causes of ALI/ARDS, and Lipopolysaccharide (LPS), the major component of the outer membranes of gram-negative bacteria, can cause lung injury and induce inflammatory response [3]. LPS-induced ALI/ARDS in mice has become a recognized model for studying ALI/ARDS, because it simulates pathological events such as inflammation and histological changes observed in this disease [4]. The receptor for advanced glycation end products (RAGE) is a transmembrane pattern recognition receptor that is expressed in mammals and belongs to the immunoglobulin (Ig) superfamily [5]. It is strongly expressed in AECs and acts as a marker of epithelial injury [6]. As an innate immune sensor, RAGE can recognize microbial pathogen-associated molecular patterns, including bacterial LPS [7]. RAGE/NF-κB signaling has been reported to be involved with LPS-induced inflammatory lung injury [8]. Besides, RAGE knockout mice displayed a notable improvement in alveolar fluid clearance and pulmonary vascular albumin leakage in response to LPS [9]. The RAGE inhibitors FPS-ZM1 and Azeliragon were found to be effective in repairing LPS-induced airway epithelium damage [10]. The main soluble forms of RAGE lack a transmembrane domain, referred to as soluble RAGE (sRAGE), has good diagnostic utility and is associated with lung injury severity in clinical settings. Elevated plasma sRAGE was associated with higher mortality in ARDS patients [11]. Nevertheless, different research has shown that administering sRAGE dramatically improved LPS-induced mortality by reducing tissue damage and cytokine release in mice models [12]. Recombinant human RAGE has been demonstrated to reduce lung edema, neutrophil infiltration, and inflammatory factors secretion in a rat model of LPS-induced ALI [13]. Although RAGE has been investigated in various pulmonary diseases and maybe a critical regulator of pulmonary inflammatory responses [14], the current research findings on RAGE are inconsistent. Moreover, in ALI/ARDS models, the alveolar epithelium suffers more direct damage due to primary pulmonary infection. Therefore, the role of RAGE in the alveolar epithelium needs further exploration.

Autophagy in AECs has attracted increasing attention, particularly with respect to lung injury caused by pneumoconiosis and nanoparticles [15, 16]. However, there is a paradoxical role of autophagy in lung injury caused by acute infection or inflammation. In previous research, autophagic proteins were decreased under LPS stimulation via activation of mammalian target of rapamycin (mTOR) signals in the mouse airway epithelium and human bronchial epithelial cells, overactivation of autophagy or genetic knockdown of mTOR significantly decreased the release of interleukin (IL)-6 and IL-8 [17]. Conversely, a recent study suggested that LPS-induced autophagy activation in AECs and inhibiting autophagy protects from inflammatory injuries [18]. These studies showed that autophagy in AECs plays an important role in ALI, however, the underlying mechanism remains for further research. Evidence suggests that inhibiting RAGE exerts a therapeutic effect in ARDS animals by restoring alveolar epithelial permeability and pulmonary fluid clearance [19]. Interestingly, it was reported that RAGE mediates autophagy which participates in the pathogenesis of different lung diseases, including ALI [20]. A recent study found that the suppression of autophagy improved pulmonary epithelial cell viability and tight junctions [21]. However, the role of RAGE in epithelial cell autophagy remains unclear.

This study, therefore, aimed to investigate the potential role of RAGE in the regulation of autophagy in ATII cells of ALI/ARDS models. We demonstrated that autophagy activation in ATII cells was changed in a time-dependent manner, and excessive autophagy intensified cell death in a RAGE-dependent manner. To the best of our knowledge, this is the first study to demonstrate that RAGE is a critical mediator of the autophagy activation of ATII cells in response to endotoxins.

## Methods

### Experimental ALI animal models

Male C57BL/6 mice (8–9 weeks old) were purchased from the Shanghai Model Organisms Center (Shanghai, China) and randomly assigned to the groups. In the laboratory, the mice were kept in a ventilated cage on a 12 h/12 h light–dark cycle at 25 °C with a humidity of 45–55%. The mice had free access to food and water. ALI/ARDS was induced in mice by intratracheal injection of LPS (10 mg/kg, *Escherichia coli* 0111:B4, Sigma-Aldrich Co., St. Louis, MO, USA). Male mice were anaesthetized by intraperitoneal (i.p.) injection with 1% pentobarbital sodium solution (50 mg/kg, Biyuntian Institute of Biotechnology, Haimen, China). The thoraxes of mice were subjected to a vertical 0-degree single-field irradiation of chest X-ray.

To obtain the best modelling, the mice were euthanized at 0, 2, 6, 12, 24, and 48 h after LPS treatment. To explore the effect of autophagy inhibition, mice were treated with 3-MA (35 mg/kg, Sigma-Aldrich Co., St. Louis, MO, USA, i.p.) for 8 h before LPS treatment. To explore whether autophagy occurs in a RAGE-dependent pathway, *Ager* gene knockout mice (*Ager*^−/−^ mice) and C57BL/6 mice were treated with or without LPS for 24 h before euthanization. The *Ager* gene in mice is equivalent to the RAGE gene in humans. The *Ager*^−/−^ knockout mice were purchased from Cyagen Biosciences Inc. Knockout of the *Ager* gene was validated by genotyping. The following polymerase chain reaction (PCR) primer pairs were used for genotyping: Primers1: (Annealing Temperature 60.0 °C), F1: 5’-GAGGTCTCCATTCTTTCTCCAGGTG-3’, R1: 5’-GTGCACACATCTGCAGAGCCAAC-3’. Primers2: (Annealing Temperature 60.0 °C), F1: 5’-GAGGTCTCCATTCTTTCTCCAGGTG-3’, R2: 5’-CTGGGATTGACTCTTGCCTCCCTC-3’. Wildtype allele had two bands with 414 bp and 717 bp, homozygotes had a PCR length of about 520 bp, and heterozygotes had three bands with 520 bp, 414 bp and 717 bp. Serum, lung tissues and bronchoalveolar lavage fluid (BALF) were collected. The lung was lavaged three times in 250 µL phosphate-buffered saline (PBS) and retrieved. The collected supernatants were stored at − 80 °C for further analysis and all experiments were repeated more than three times.

### Cell culture and treatments

Human ATII cells, namely, A549 cells were obtained from the Chinese Academy of Science. Primary human ATII cells were purchased from iCell Bioscience, Inc. (Shanghai). Cells were seeded into 6-well plates and cultured in Dulbecco’s-modified Eagle’s medium (for A549 cells) or epithelial cell complete medium (for primary ATII cells) supplemented with 10% fetal bovine serum, 100 U/mL penicillin and 100 μg/mL streptomycin at 37 °C in a humidified atmosphere containing 5% CO2. Cells were grown until 70% confluence before drug treatments were performed. Cells were treated with LPS (10 μg/mL) for 0, 12, 24, and 36 h and LPS (0, 1, 10, 100 μg/mL) for 24 h. The cell samples were harvested at the appointed time after the addition of LPS to further analysis. 3-MA was used at a dose of 5 mM for 12 h before LPS treatment (10 μg/mL). RAGE small interfering RNA (siRAGE) transfection was incubated for 48 h before LPS treatment (10 μg/mL). All transfection kits and reagents were purchased from Guangzhou Ribo Biology Co., Ltd (China). In the Additional files 1, 2, to address the roles of sRAGE, we pretreated A549 cells with or without the recombinant human protein which lacking signal peptide (C423, 25 μg/mL, novoprotein Technology CO., LTD, Suzhou, China) before LPS stimulation.

### Cell counting Kit-8 (CCK-8) assay

CCK-8 assay (Biyuntian Institute of Biotechnology, Haimen, China) was performed to determine cell viability. Cells were seeded into 96-well plates (1 × 10^6^ cells/well) and cultivated at 37 °C under 5% CO_2_ atmosphere. The cell suspension was mixed with 10 μL CCK-8 solution for 1 h and the optical density of each well was assessed at 450 nm using a microplate reader (Thermo Fisher Scientific, MA, USA).

### Enzyme-linked immunosorbent assay (ELISA)

The sRAGE concentration in BALF was determined by double antibody sandwich ELISA (MultiScience LIANKE Biotech, CO., LTD, Hangzhou, China). IL-8 and IL-6 levels in BALF were determined with commercially available ELISA kits (MultiScience LIANKE Biotech, CO., LTD, Hangzhou, China) according to the manufacturer’s instructions. The optical density at 450 nm was measured using a microplate reader (Thermo Fisher Scientific, MA, USA).

### Histological analysis

The right lungs of mice were collected and fixed in 4% paraformaldehyde buffer overnight at room temperature, then dehydrated and embedded in paraffin, sectioned into 4-µm slices and stained with hematoxylin and eosin (HE). The samples were photographed under a light microscope (LEICA, Leica Microsystems, Wetzlar, Germany), with at least three images acquired for each sample.

### Immunohistochemical analysis

Paraffin-embedded tissue sections were used for immunohistochemical staining. The sections were deparaffinized with 100% xylene and rehydrated in graded ethanol series, followed by antigen retrieval using citrate buffer (pH = 6) for 20 min. The rabbit polyclonal anti‐RAGE antibody (1:100; cat. no. ab37647; Abcam, Cambridge, Mass) was used for staining slides at 4 °C overnight. After washing, poly horseradish peroxidase (HRP) anti‐rabbit secondary antibody (1: 5000; cat. no. A24531, Thermo Fisher Scientific Inc. USA) was added and incubated at room temperature for 20 min. Diaminobenzidine was used as chromogen.

### Immunofluorescence staining

The paraffin sections of mouse lungs, A549 cells and primary ATII cells were used for immunofluorescence staining. The sections were incubated with rabbit anti-cleaved caspase 3 antibodies (1:1000, cat. no. #9664; Cell Signaling Technology, MA, USA), anti-RAGE (1:1000, ab37647; Abcam, Cambridge, Mass.), and rabbit IgG isotype (1:1000, ab199093; Abcam, Cambridge, Mass.) at 4 °C overnight, followed by incubation with fluorescently labelled secondary antibodies (1:100, Invitrogen, Carlsbad, CA). Images were acquired using a panoramic confocal camera (3DHistech, Budapest, Hungary).

For immunofluorescence double staining, the mouse lung sections prepared as above were exposed to anti-RAGE (1:1000, ab37647, Abcam, Cambridge, Mass.) overnight at 4℃. After washing with PBS three times, the slides were incubated with fluorescently labelled secondary antibodies at room temperature in the dark for 50 min, followed by incubation with CY3-TSA at room temperature in the dark for 10 min and washing with PBS three times. Subsequently, microwave treatment was performed on the sections to remove the primary and secondary antibodies. The sections were then exposed to anti-proSP-C (1:25, AP12333A, Abcepta, Beijing, China) or anti-HOPX (1:500, 11419-1-AP, Proteintech, Wuhan, China) and processed as described above, except that they were incubated with FITC-TSA. DAPI was used for staining for 5 min and the sections were blocked by antifluorescence quenching sealing liquid. The expression and coexpression of RAGE and proSP-C or HOPX were examined under a microscope (Nikon Eclipse C1, Nikon Instruments Inc., Melville, NY), photographed using a Nikon DS-U3 camera, and then evaluated by two pathologists. RAGE protein exhibited red fluorescence (labelled with CY3) while proSP-C or HOPX protein exhibited green fluorescence (labelled with FITC). The cell nucleus exhibited blue fluorescence (stained with DAPI). In addition, terminal deoxynucleotidyl transferase dUTP nick-end labelling (TUNEL) assay was performed to detect apoptosis (Roche, Mannheim, Germany) according to the manufacturer’s protocol.

### Transmission electron microscopy

Fresh lungs were separated and cut into 1-mm cubes immediately, which were then fixed by immersion in 2.5% glutaraldehyde buffer for 24 h, followed by washing thrice with PBS (15 min each time). The samples were then fixed in 1% osmium tetroxide for 2 h, dehydrated in fractionated ethanol solution (50%, 70%, 80%, 90%, 95%, 100%, and 100%), and embedded in epoxy resin. Ultrathin sections (60–80 nm) double-stained with a saturated aqueous solution of 2% uranyl acetate and lead citrate were examined under a transmission electron microscope (TECNAI G2 F20, Fei, USA) and images were acquired.

### Western blotting analysis

Western blotting was performed as previously described [22]. Anti-RAGE (1:1000, #6996S; Cell Signaling Technology, Inc., MA, USA), anti-STAT3 (1:1000, #12640; Cell Signaling Technology, Inc., MA, USA), anti-phosphorylated STAT3 (1:2000, #9145; Cell Signaling Technology, Inc., MA, USA), anti-cleaved caspase 3 (1:1000, #9664; Cell Signaling Technology, Inc., MA, USA), anti-LC3II/I (1:1000, #4108; Cell Signaling Technology, Inc., MA, USA), anti-Beclin1 (1:1000, #3495; Cell Signaling Technology, Inc., MA, USA) and anti-GADPH (1:1000, #2118; Cell Signaling Technology, Inc., MA, USA) were used as primary antibodies. The membranes were incubated with primary antibodies at 4℃ overnight, followed by incubation with HRP‐conjugated secondary antirabbit antibody (1:50,000; cat. no. BM2006; BOSTER Biological Technology Co., Ltd., Wuhan, Hubei, China) at 37 °C for 1 h after washing. Anti-GADPH was used as an endogenous control for other proteins. Images were obtained using a chemiluminescent western blot scanner.

### Statistical analysis

Differences in data were compared using one‐way analysis of variance. Data were analyzed using the GraphPad Prism 11 software (GraphPad Software, Inc., La Jolla, CA) or SPSS 23.0 (IBM SPSS, Armonk, NY). A P value of < 0.05 was considered statistically significant.

## Results

### Activated RAGE signals and inflammatory responses in LPS-induced lung injury

**Fig. 1:**
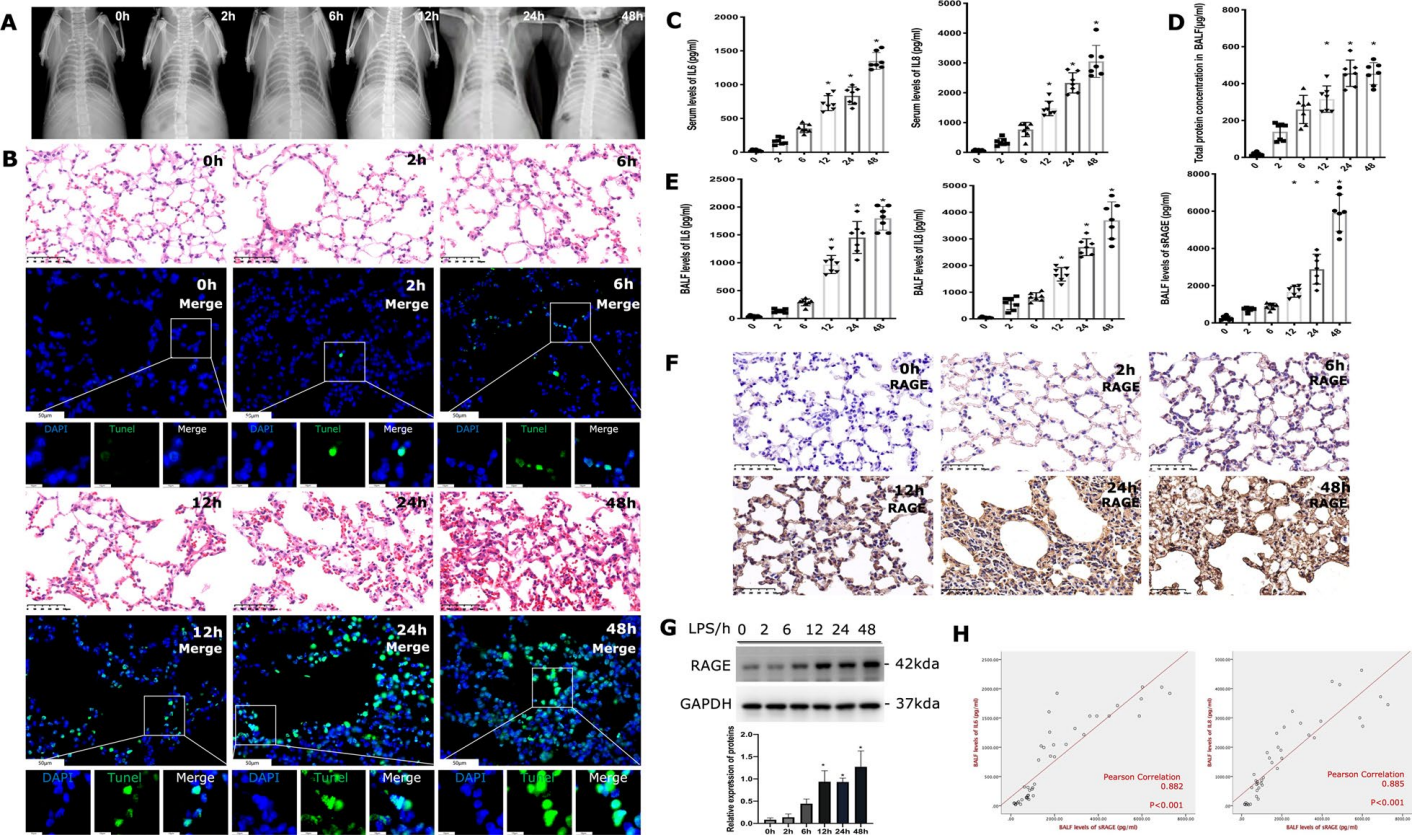
Activated RAGE signals and increased inflammation is characteristic of LPS-induced lung injury. **A** Chest radiograph of mice treated with LPS through nasal inhalation. **B** Slices of mice lungs were prepared. Representative HE staining images of mouse lung sections showing injury after LPS stimulation at different time points, images × 40 magnification, scale bar 50 μm. The apoptosis of lung cells was evaluated by the TUNEL assay and images were obtained by spinning disk confocal microscopy at × 40 magnification (scale bar 50 μm) and × 200 magnification (scale bar 10 μm). **C** Serum IL-6 and IL-8 concentrations were determined by indicated ELISA kit. **D** The total protein levels of BALF in LPS-induced lung injury mice. **E** The levels of IL-6, IL-8 and sRAGE in BALF of mice upon LPS treated at different time points. **F** Immunochemistry of RAGE expression in the mice lung sections under the stimulation of LPS. Images × 40 magnification, scale bar 50 μm. **G** The immunoblot of RAGE in lung tissue was performed and its expression increased over time, n ≥ 3 examinations. **H** The relationship of IL-6 and IL-8 with sRAGE in the BALF was performed by Pearson’s correlation analysis. *Indicates the significant difference compared with the control group (0 h), p < 0.05, differences in characteristics between groups were analyzed using the Kruskal–Wallis test with Dunn’s post hoc tests

To analyse the pulmonary effusion of mice, we have shot radiography of them. The X-ray results were characterized by interstitial exudative inflammation of the lung with formation in the acute phase and aggravation with time (Fig. 1A). HE sections of mice lungs treated with LPS for 6 h to 48 h were characterized by alveolar structure destruction, edema and thickening of the alveolar wall, inflammatory infiltration and focal necrosis in a time-dependent manner (Fig. 1B). We further detected apoptotic cells of lung tissues by TUNEL assay, which showed an increase in apoptosis rate as time progressed (Fig. 1B). Meanwhile, the serum (Fig. 1C) and BALF (Fig. 1E) levels of IL-6 and IL-8 were significantly increased in LPS-treated mice compared with the control group, and the elevated levels of IL-6 and IL-8 persisted from 2 to 48 h following LPS treatment both in serum and BALF.

Interestingly, we noticed that the total proteins in BALF were increased gradually after LPS treatment (Fig. 1D), indicating proteins leakage into the alveolar space. To investigate the function of RAGE in acute inflammatory lung injury, we detected the levels of sRAGE in BALF and RAGE in lung tissues respectively. As a result, sRAGE in BALF was gradually increased upon LPS administration and was significantly higher than in control (0 h) after 24 h (Fig. 1E). The same tendency was observed in the expression of RAGE in lung tissues (Fig. 1F detected by immunohistochemistry; Fig. 1G detected by western blot). As shown in Fig. 1E, BALF levels of IL-6 and IL-8 were significantly increased compared with controls. Pearson’s correlation analysis showed that levels of sRAGE were positively correlated with IL-6 and IL-8 (Fig. 1H).

**Fig. 2:**
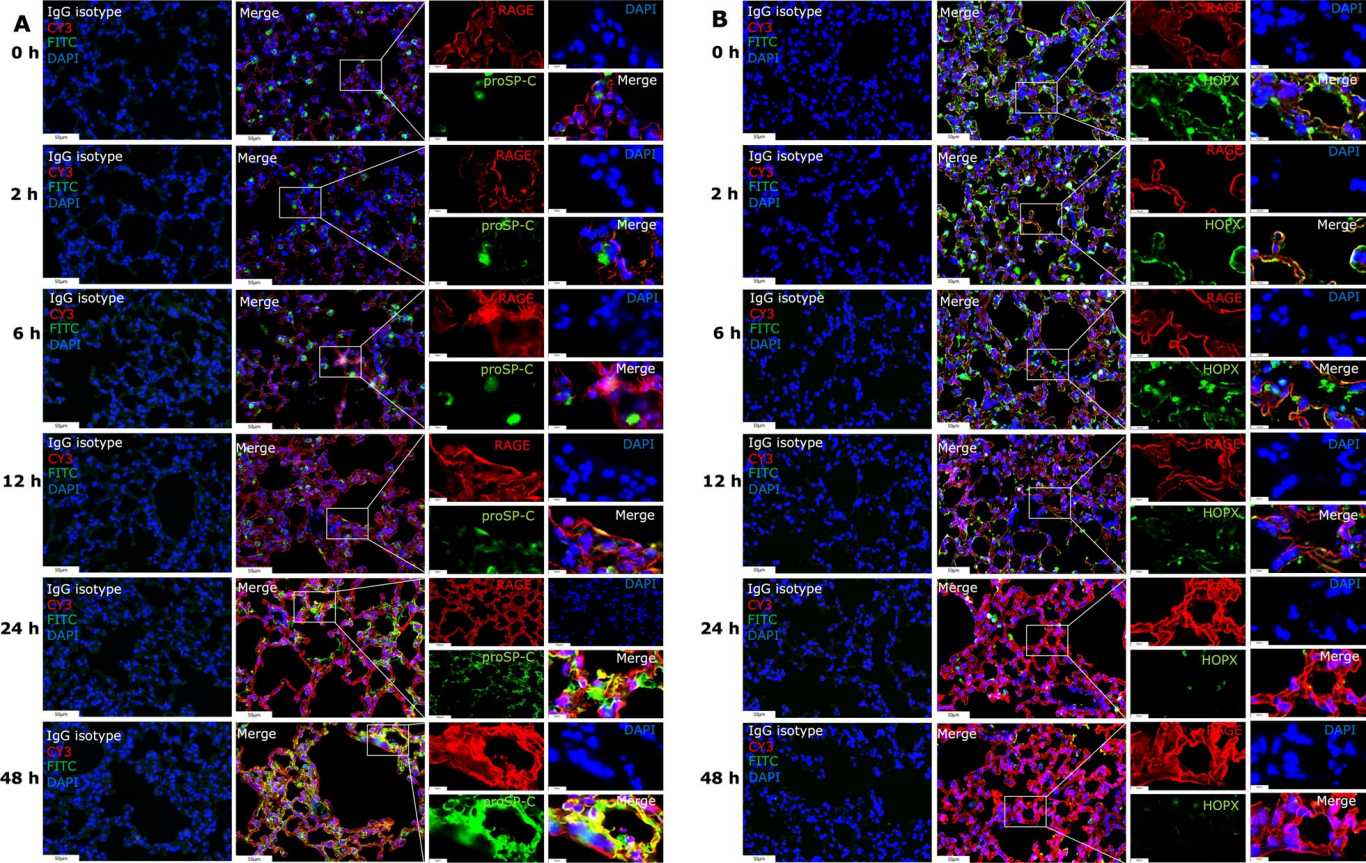
RAGE signal activation varies between type I and type II AECs in LPS-treated lungs. **A** Representative images of co-localization patterns of RAGE and proSP-C (ATII marker) in lung tissues using multiplexed immunofluorescence staining; **B** Co-localization patterns of RAGE and HOPX (ATI marker) in lung tissues using multiplexed immunofluorescence staining (images × 40 magnification, scale bar 50 μm, Images × 200 magnification, scale bar 10 μm), RAGE protein showed red fluorescence (labelled by CY3), and ProSP-C or HOPX protein showed green fluorescence (labelled by FITC). The cell nucleus showed blue fluorescence (stained by DAPI)

Additionally, the localization of RAGE with proSP-C, a hallmark of ATII cells, co-localized and steadily increased in response to LPS treatment (Fig. 2A). While RAGE expression was reduced when co-localized with the ATI cell marker HOPX (Fig. 2B). According to the changes of HOPX expression at the indicated time point of LPS treatment, we speculate that the decreased expression of RAGE in ATI cells could be due to LPS-induced apoptosis. In another aspect, these results suggest that ATII cells express RAGE more frequently under LPS stimulation, the increased expression of RAGE and proSP-C implies the proliferation of ATII cells during LPS-induced lung injury, which may contribute to the overall elevated RAGE levels in the lungs. Collectively, LPS insulting enhanced the production and release of RAGE and led to a breakdown of lung tissue by elevated inflammatory responses.

### Autophagy was persistently activated in LPS-induced lung injury

**Fig. 3:**
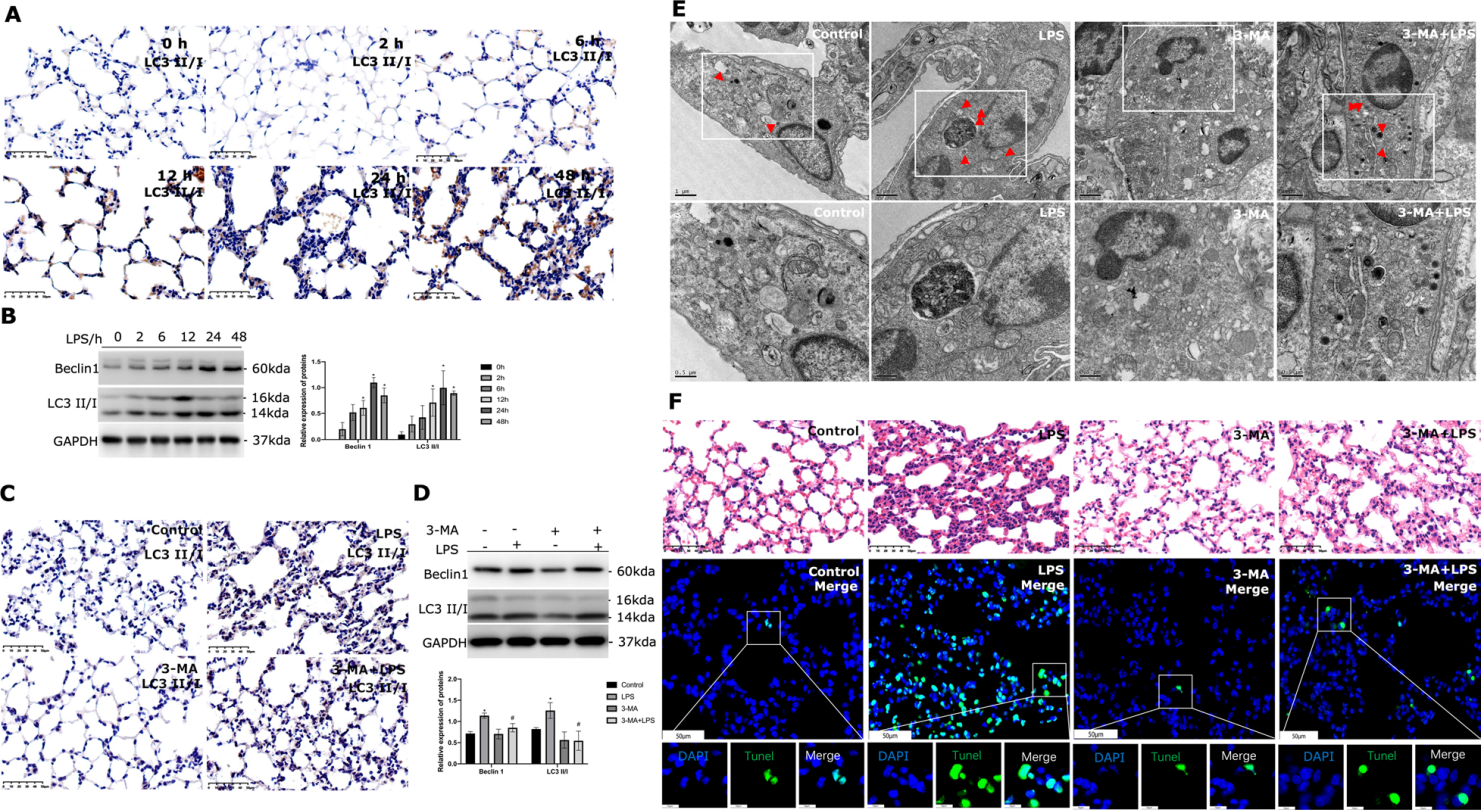
Increased activation of autophagy in LPS-induced lung injury. **A** LC3 II/I expression in lung tissues under LPS stimulation for different hours and Immunochemistry was performed with LC3 II/I antibody. Images × 40 magnification, scale bar 50 μm. **B** Immunoblots of Beclin1 and LC3II/I were performed in mice lungs upon LPS treated at the indicated time, n ≥ 3 examinations. **C** Immunochemistry of LC3 II/I expression in lung tissues upon LPS treated when being pretreated with or without 3-MA. Images × 40 magnification, scale bar 50 μm. **D** The protein levels of Beclin1 and LC3II/I in mice lungs upon LPS treated when being pretreated with or without 3-MA performed by western blotting, n ≥ 3 examinations. **E** Transmission electron microscopy was used to observe the autophagic body. Red arrows indicate the number and location of autophagosomes. The scale bar for original images is 1 µm; the scale bar for enlarged images is 500 nm. **F** HE staining images of mouse lung sections (images × 40 magnification, scale bar 50 μm). The apoptosis of lung cells was evaluated. The lung sections were fixed and immunostained by the TUNEL assay and caught images by spinning disk confocal microscopy at × 40 magnification (scale bar 50 μm) and × 200 magnification (scale bar 10 μm). * Indicates the significant difference compared with the Control group, # indicates the significant difference compared with the LPS group. P < 0.05, differences in characteristics between groups were analyzed using the Kruskal–Wallis test with Dunn’s post hoc tests

We investigated the role of autophagy in LPS-induced lung injury. Immunohistochemistry revealed the accumulation of LC3 II/I (Fig. 3A), and lung tissues from mice exposed to LPS showed the increased expression of autophagy-related proteins, such as LC3 II and Beclin1 (Fig. 3B). These findings suggested that autophagy was gradually activated in the course of LPS-induced lung injury. When mice were given 3-MA prior to inhaling LPS, these responses were dramatically suppressed, regardless of whether they were seen by immunohistochemistry (Fig. 3C) or western blotting (Fig. 3D). Additionally, transmission electron microscopy demonstrated that 3-MA treatment reduced autophagosomes (Fig. 3E). Inhibiting autophagy by pretreating with 3-MA altered the lung injury response in HE sections of LPS treated mice lungs as compared to the control group (Fig. 3F). Additionally, the TUNEL assay showed that autophagy suppression reduced the rate of apoptosis in the lungs (Fig. 3F).

### RAGE activation contributes to autophagic cell death in LPS-induced lung injury

**Fig. 4:**
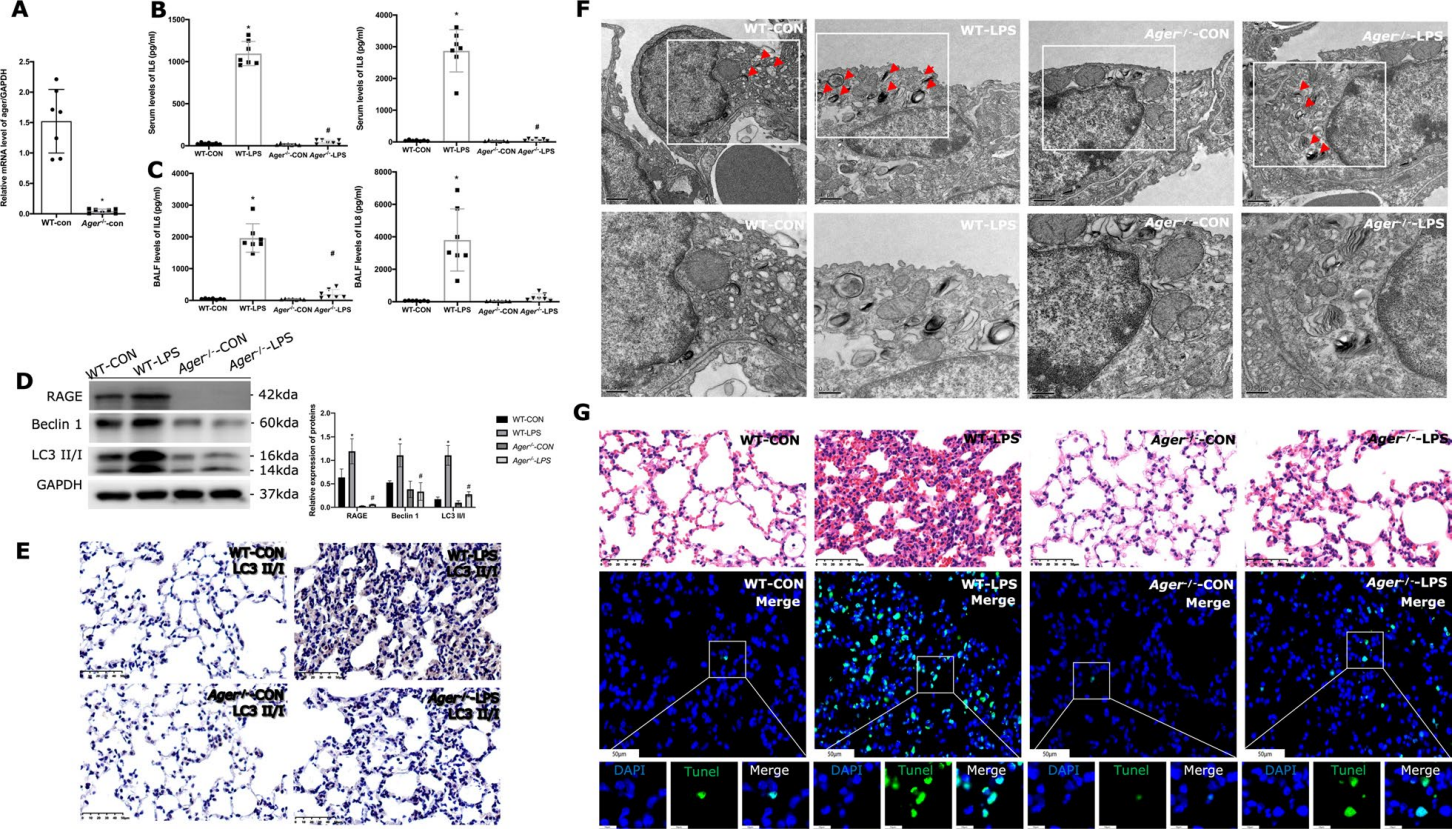
LPS-induced autophagic cell death is related to RAGE activation. **A** *Ager* mRNA in WT and Ager^−/−^ mice lungs. **B** Serum levels of IL-6 and IL-8 in WT and *Ager*^−/−^ mice. **C** Levels of IL-6 and IL-8 in BALF of WT and *Ager*^−/−^ mice. IL-6 and IL-8 were determined by commercial ELISA kits (n = 7). **D** The immunoblot of RAGE in the lung sections of WT and *Ager*^−/−^ mice (n ≥ 3 examinations). **E** Immunochemistry of LC3 II/I expression in lung sections. Images × 40 magnification, scale bar 50 μm. **F** Mice lung sections were processed for transmission electron microscopy to observe the autophagic body. Red arrows indicate the number and location of autophagosomes. The scale bar for original images is 1 µm; the scale bar for enlarged images is 500 nm. **G** HE staining of lung sections from WT mice or *Ager*^−/−^ mice under LPS stimulation. The apoptosis of lung cells was evaluated by the TUNEL assay and images were obtained by spinning disk confocal microscopy at × 40 magnification (scale bar 50 μm) and × 200 magnification (scale bar 10 μm). P-values by Kruskal–Wallis test with Dunn’s post hoc tests. * indicates the significant difference compared with WT-CON group, # indicates the significant difference compared with WT-LPS group

We used *Ager*^−/−^ mice models to simulate acute lung injury in order to more thoroughly analyze the involvement of RAGE in autophagy activation upon LPS stimulation. The levels of *Ager* mRNA and protein were found to be much lower in the lung tissues of *Ager*^−/−^ mice (Fig. 4A, D), confirming a total type of homozygous mice with *Ager* gene deletion. Lower levels of IL-6 and IL-8 have emerged in *Ager*^−/−^ mice compared with wild-type mice no matter in serum (Fig. 4B) and BALF (Fig. 4C). Therefore, we speculate that activated RAGE signals promote the release of inflammatory substances and aggravate lung injury.

Our initial experiments demonstrated that LPS significantly up-regulated the level of RAGE and autophagy was consistently activated in the lungs of mice. To determine whether RAGE induced autophagy contributes to inflammatory lung injury evoked by LPS, we detected autophagy-related proteins of Beclin1 and LC3 II/I in *Ager*^−/−^ mice. Beclin1 and the LC3 II to GAPDH ratio were higher in the lung tissues of *Ager*^−/−^ mice compared to wild-type mice (Fig. 4D). Immunohistochemistry revealed that *Ager*^−/−^ mice had lower levels of LC3 II/I expression in their lung tissues than wild-type mice did (Fig. 4E). Furthermore, transmission electron microscopy revealed that whether or not the mice were exposed to LPS, *Ager*^−/−^ mice had fewer autophagosomes than wild-type mice (Fig. 4F). As a result of LPS stimulation, alveolar structural breakdown, edema and thickening of the alveolar wall, inflammatory infiltration, and localized necrosis appeared on the lung's histology (Fig. 4G). However, *Ager*^−/−^ mice represented a slight injury than that in wild-type mice (Fig. 4G). LPS stimulation promotes cell apoptosis while TUNEL-positive cells decreased in *Ager*^−/−^ mice (Fig. 4G).

**Fig. 5:**
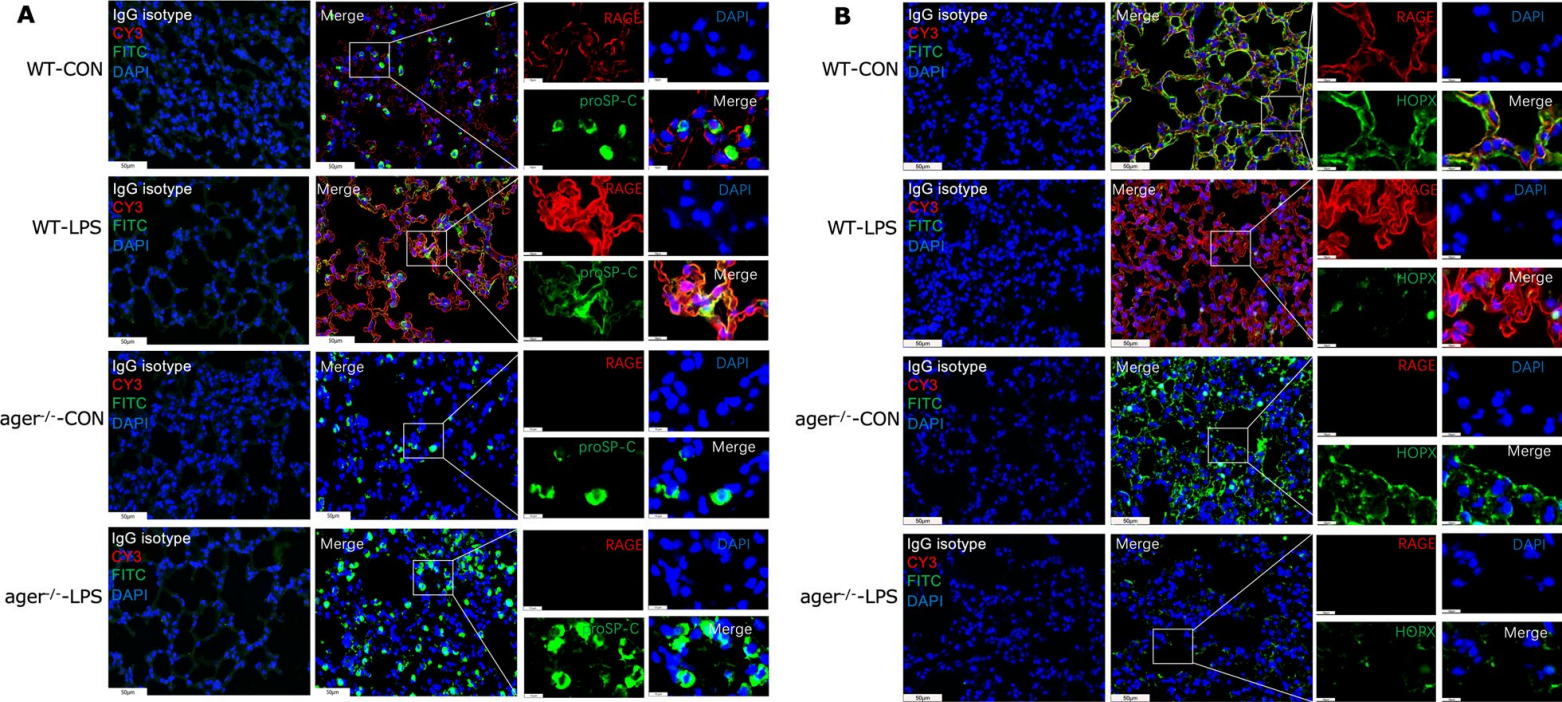
Activated RAGE signals and co-localization with proSP-C and HOPX in WT and *Ager*^−/−^ mice. Representative images of co-localization pattern of RAGE and proSP-C (**A**) or HOPX (**B**) in lung sections from WT mice or *Ager*^−/−^ mice under LPS stimulation. Images at × 40 magnification, scale bar 50 μm and images at × 200 magnification, scale bar 10 μm. RAGE protein showed red fluorescence (labelled by CY3), proSP-C and HOPX protein showed green fluorescence (labelled by FITC). The cell nucleus showed blue fluorescence (stained by DAPI)

Meanwhile, we detected the co-expression of RAGE and proSP-C or HOPX in the lungs of *Ager*^−/−^ mice and WT mice. LPS stimulation boosted the co-expression of RAGE and proSP-C (Fig. 5A). However, there was limited co-expression of RAGE and HOPX under LPS stimulation, and there was no discernible difference in HOPX expression between WT mice nor *Ager*^−/−^ mice, indicating that RAGE plays a critical role in LPS induced autophagic death. Therefore, we surmise that LPS stimulated an increase in ATII cells and partially caused autophagic cell death in a manner dependent on RAGE.

### Autophagic cell death in ATII cells injury

**Fig. 6:**
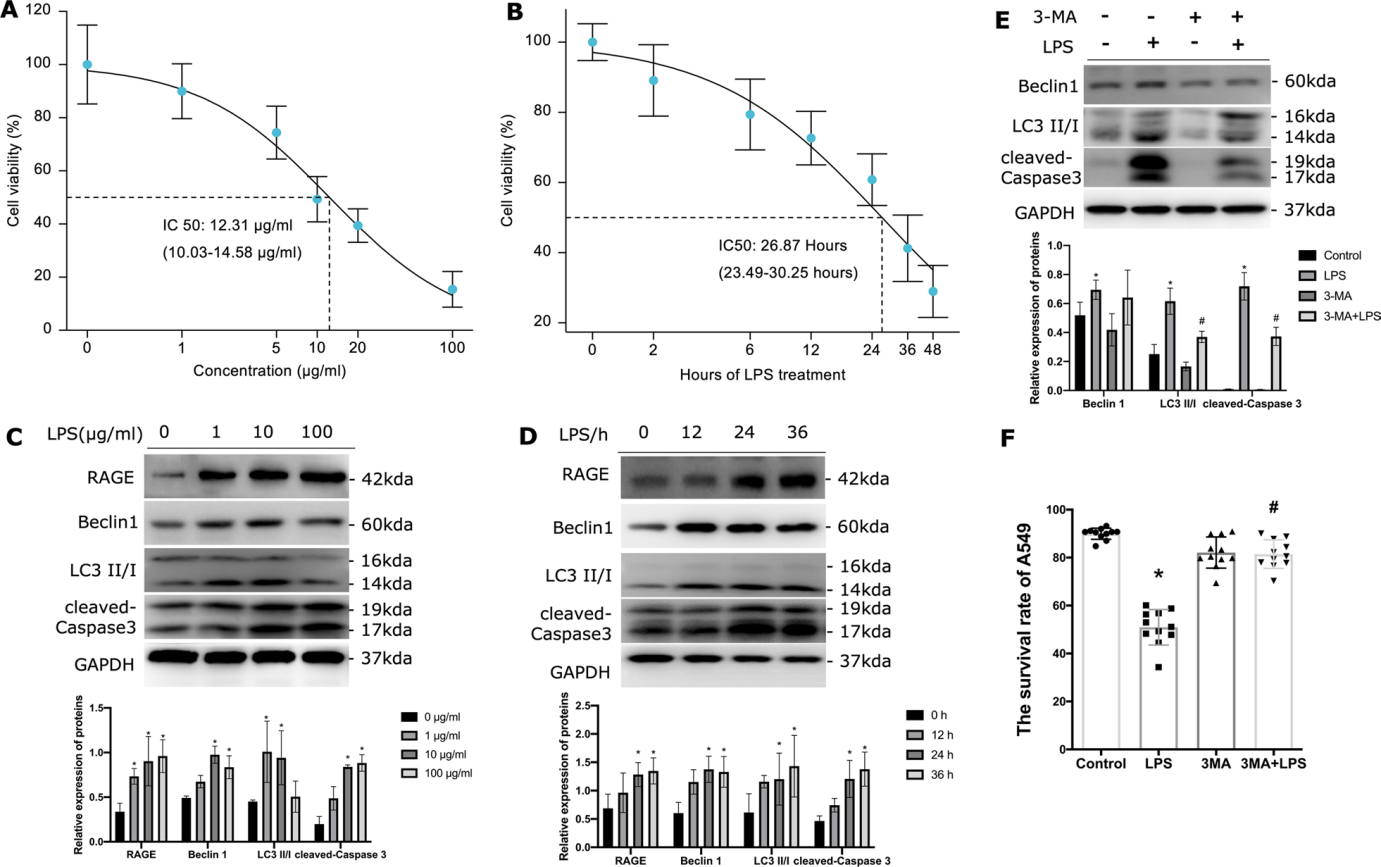
Autophagic cell death happened in A549 cells induced by LPS. The effect of LPS on A549 cells at different concentrations (**A**) and different time points (**B**) was detected by CCK-8. The immunoblot of RAGE, Beclin1, LC3II/I and cleaved Caspase 3 in A549 cells, when treated with different concentrations (**C**) and different time points (**D**) of LPS. Each experiment was repeated more than three times. **E** The immunoblot of Beclin1, LC3II/I and cleaved Caspase 3 in A549 cells upon LPS treated when being pretreated with or without 3-MA. 3-MA effectively inhibited the autophagy activation induced by LPS treatment. **F** The cell survival rate of A549 cells in response to 3-MA pre-treatment followed by LPS stimulation. *Indicates the significant difference compared with the LPS 0 μg/mL group or LPS 0 h group or Control group. # Indicates a significant difference compared with the LPS group. P < 0.05, P-values by Kruskal–Wallis test with Dunn’s post hoc tests. each experiment was repeated more than three times

To focus on the change in ATII cells, we incubated A549 cells with different concentrations of LPS (Fig. 6A) and at different time points (Fig. 6B) to establish optimization in vitro models. A549 cells were treated with 10 μg/mL LPS for 24 h and the development of autophagy and apoptosis marker protein after LPS treatment were evaluated. The results showed that RAGE increased over time in LPS dose-dependent manner, and the proteins of autophagy marker (Beclin1 and LC3 II/I) and cell apoptosis marker (cleaved Caspase3) also increased over time (Fig. 6C, D). Beclin1 and the LC3 II/GAPDH ratio increased when the concentration of LPS was no more than 10 μg/mL (Fig. 6C). Autophagy was decreased and cell death intensified once LPS reached 100 μg/mL (Fig. 6C). These data demonstrated that autophagy can be observed in A549 cells and remained relatively active when treated with LPS (10 μg/mL) for 24 h. To confirm the potential roles of autophagy activation in alveolar epithelial injury, 3-methyladenine (3-MA) was used to inhibit autophagy in A549 cell lines. LC3-II induction was suppressed in the presence of 3-MA (Fig. 6E). Suppression of autophagy by 3-MA promotes the survival of A549 cells indicated by the decreasing levels of cleaved Caspase 3 (Fig. 6E). Moreover, the cell viability was significantly increased after being treated with 3-MA (Fig. 6F), indicating the inhibition of excessive autophagy survival of AECs.

### Autophagy of ATII cells in a RAGE-dependent pathway under endotoxin

**Fig. 7:**
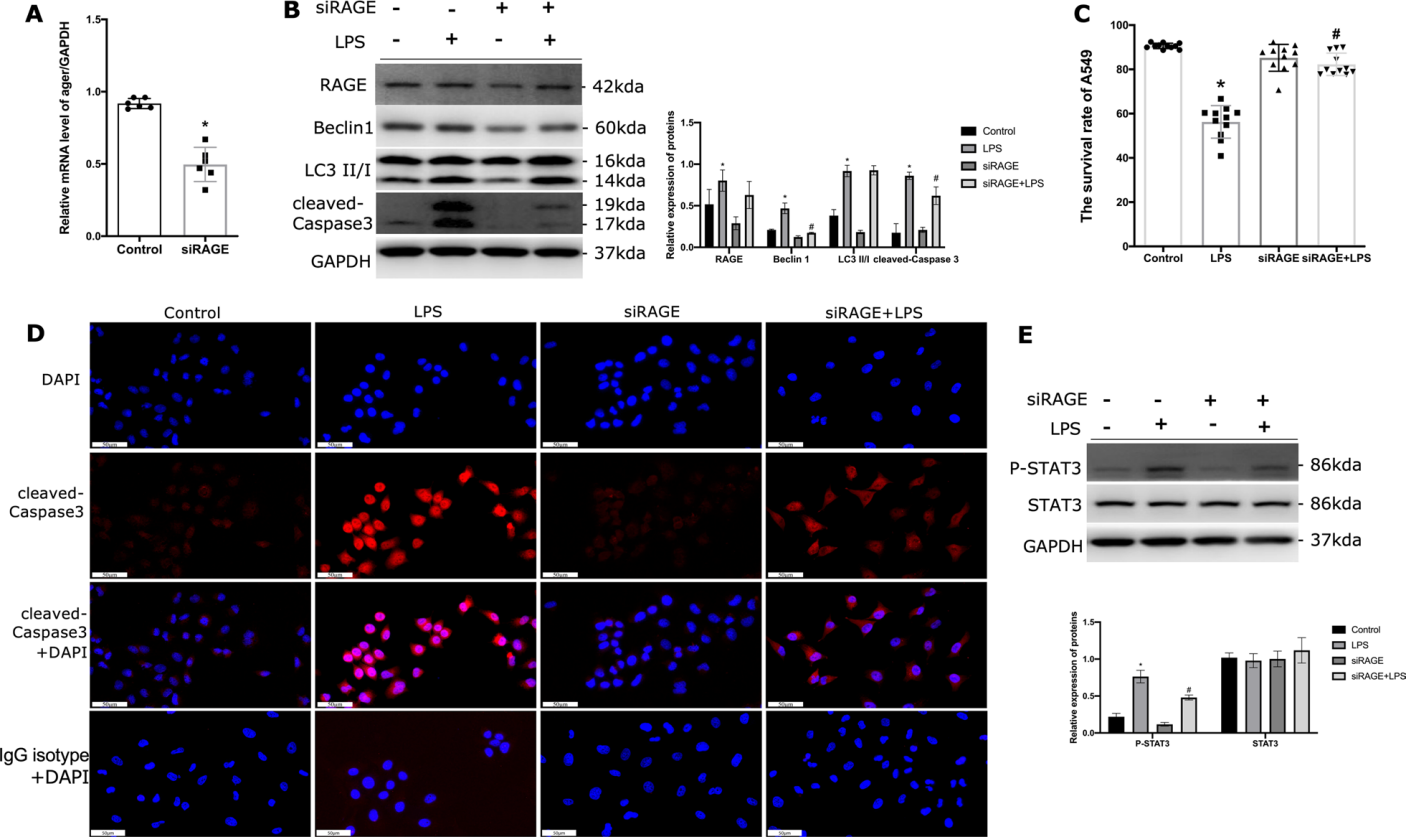
LPS-induced autophagic death in A549 cells depended on RAGE activation. **A** Treated A549 cells with siRAGE and detected the RAGE mRNA expression. The expression of *Ager* genes was decreased by 50%. **B** Detected the immunoblots of RAGE as well as Beclin1, LC3 II/I and cleaved Caspase 3 in A549 cells, each experiment was repeated more than three times. **C** The cell survival rate of A549 cells in response to siRAGE pre-treatment followed by LPS stimulation. Data were obtained from CCK-8 experiments and performed more than three times. **D** Immunofluorescence of cleaved Caspase3 to detect the apoptosis of A549 cells, spinning disk confocal microscopy at × 40 magnification (scale bar 50 μm). **E** Detected the immunoblots of STAT3 and phosphorylation STAT3 (p-STAT3) in A549 cells, each experiment was repeated more than three times. *Indicates the significant difference compared with the control group. # Indicates the significant difference compared with LPS group. P < 0.05, differences in characteristics between groups were analyzed using the Kruskal–Wallis test with Dunn’s post hoc tests

Considering autophagy activation in ATII cells is accompanied by an increase of RAGE, RAGE was knocked down via siRAGE. The expression of *Ager* mRNA was significantly suppressed in A549 cells (Fig. 7A), corresponding to the expression of RAGE protein was decreased (Fig. 7B). Moreover, the expression of LC3 II formation and Beclin1 was significantly decreased in the group of RAGE knockdown, the reduction of RAGE decreased the protein levels of cleaved Caspase3 (Fig. 7B). Consequently, the effects of RAGE induced overexpression of autophagy and exacerbated cell apoptosis upon LPS were blocked. The above results observed in A549 cell line were similar to those in primary ATII cells (Additional file 1: Fig. S1A–D). To further address the differing roles of sRAGE and mRAGE, we pretreated A549 cells with or without sRAGE before LPS stimulation. It was not found that sRAGE could effectively induce autophagy and reduce cell apoptosis (Additional file 2: Fig. S2A,B).

Cell viability was significantly increased when RAGE was knocked down compared with the control group treated with LPS (Fig. 7C). A reverse change of cleaved Caspase3 detected by immunofluorescence was also discovered in the siRAGE treated group (Fig. 7D). A previous study shows that STAT3 activation exacerbates inflammatory responses in mammal pulmonary [23]. In this study, the phosphorylated STAT3 (P-STAT3) levels were significantly increased in response to LPS stimulation, while STAT3 activation in siRAGE cells was noted to be reduced (Fig. 7E).

## Discussion

This study aimed to examine the role of RAGE induced autophagy in ATII cells in response to endotoxin stimulation. In this study, an increase of autophagy activity in the lung, particularly in ATII cells, was observed, which was associated with RAGE signal activation in ALI. Specific knockdown of RAGE improved LPS-induced ATII cells dysfunction and reduced autophagy activation, implying a harmful role of autophagy in pro-inflammatory cytokine-induced ALI/ARDS. Our findings suggest that the overactivation of autophagy via a RAGE-dependent pathway contributes to ALI upon endotoxin exposure. To the best of our knowledge, this is the first study to demonstrate that epithelial cell autophagy activation under LPS stimulation occurred via a RAGE-dependent pathway.

Type I and type II AECs together make up the epithelium of alveoli and play critical roles in lung physiology and pathology. The alveolar epithelium provides protection against environmental insults, regulates water and ion transport and produces pulmonary surfactants to maintain alveolar homeostasis [24]. Whether the injury of type I alveolar epithelial cells, which account for more than 90% of the alveolar area, or the injury of other cells in the lungs or vascular endothelium, they are the factors of the occurrence and development of ARDS [25]. Alveolar epithelial damage impairs surfactant release in primary ARDS, inhibits alveolar fluid clearance, and lowers lung compliance [9]. As shown in our results, when type I cells are lost due to lung injury, type II cells act as progenitor cells, multiply and replace those cells. And that plays a crucial part in epithelial healing and the development of lung fibrosis in ARDS [2]. Therefore, protecting AECs from damage is a potential therapeutic strategy against ALI.

In the present study, we found that injury of the alveolar epithelium was accompanied by autophagy enhancement and we observed that overexpression of autophagy-related factors resulted in apoptosis of ATII cells. Although autophagy was initially described as a non-apoptotic pathway of programmed cell death, it now appears that autophagy plays a highly context-specific part in mediating cell death [26]. Activation of autophagy may be a common signaling mechanism in the pathogenesis of ALI. This was supported by a study suggesting that autophagy was activated in LPS-induced ALI mice models [27]. Autophagic cell death in the lungs is an acute phase response that contributes to the development of ARDS [28]. Levels of autophagy proteins (Beclin1 and LC3 II) and inflammatory factors (IL-6 and TNF- α) were all increased in mice AEC cells under endotoxin stimulation, confirming that autophagy is a way of stress response of lung epithelial cells [29]. In vitro*,* it was shown that LPS may lead to autophagic death of A549 cells through PERK-dependent unfolded protein reactions (UPR) [30]. Consistently, the autophagy in ATII cells was correlated with the stimulation intensity of LPS in our study; however, when 100 µg/mL LPS was used, the activity of autophagy in ATII cells was decreased, but apoptosis was still evident. It was considered that autophagy was further uncontrolled and that it exacerbated cell damage. Whether in inhalation lung injury mouse models or LPS-treated A549 cells or LPS-treated primary ATII cells, when autophagy activity was inhibited by 3-MA, the vigor of cells was recovered, the apoptosis of cells and lung tissue injury was reduced compared with the uninhibited group, suggesting that uncontrollable autophagy led to AECs damage and subsequently developed into ALI or even ARDS; inhibition of autophagy activity may be an effective measure to protect from lung injury. Remarkably, we noted that autophagy activation under endotoxin stimulation was correlated with the RAGE/STAT3 pathway, and mapped directly to the ability of ATII cells to survive cytotoxic insult, suggesting that RAGE may be a potential mediator of enhanced autophagy in the inflammation microenvironment.

ALI/ARDS secondary to pulmonary infection developed in the stepwise process of the overwhelming inflammatory response [12]. As a receptor of many proinflammatory ligands, the role assigned to RAGE is to initiate the inflammatory response, which is primarily based on the following findings: first, RAGE is highly expressed in inflammatory lesions and produces pro-inflammatory mediators in numerous inflammatory illnesses; second, blockage of RAGEs restrained inflammatory response by arresting central inflammation signaling pathways [31]. In patients with infection-related ARDS, it was found that both serum and BALF levels of sRAGE were much higher than those in control subjects, and they were positively correlated with levels of IL-6 and IL-8 [9], which was consistent with our findings. In addition, another study revealed that sRAGE enhanced IL-6 release in the absence of S100B in alveolar type I-like cells and that sRAGE could have proinflammatory properties [32]. sRAGE has been traditionally considered a sink for proinflammatory RAGE ligands and as such has been associated with protection from inflammatory stress and disease [5]. In addition to behaving as a decoy receptor, sRAGE may transduce proinflammatory signals, thereby inducing leukocyte recruitment to sites of injury or inflammation [33]. High levels of sRAGE in circulation indicated that cell surface RAGE has been overstimulation, which if it persists might intensify proinflammatory processes and exacerbate pathological states [34]. Consistent with this role, the bacterial burden and neutrophil infiltration was shown to worsen following sRAGE administration in a mouse model of bacterial lung infection, indicating that sRAGE may indeed sustain inflammation in acute settings [35]. However, despite having a positive connection with inflammatory factors in LPS-induced mice, we found that sRAGE could not superimpose on LPS-induced autophagy and apoptosis in ATII cells. So, further investigation is required to fully comprehend the intricate biological characteristics of sRAGE on different types of AT cells and their contribution to LPS-induced inflammatory response. In addition, we found that RAGE deficiency significantly reduced pulmonary inflammatory infiltration and pulmonary edema either by reducing cytokine release or by inhibiting autophagy activity. In general, LPS is frequently used to induce sepsis [18]. Intriguingly, a study reported that the increased serum levels of sRAGE are associated with ALI but not sepsis or septic shock, suggesting that RAGE is a biomarker of lung injury rather than sepsis [36]. It was verified that *Ager*^−/−^ mice were also partially protected from injury following gram-negative (*E. coli*) or gram-positive (*Streptococcus pneumoniae*) bacterial challenges [37, 38]. Thus, we speculate that RAGE is the key mediator of ATII cells injury underlying inflammation. In this study, LPS induced the activation of RAGE and autophagy, accompanied by STAT3 phosphorylation, and RAGE deletion leads to weakened activation of downstream protein STAT3. STAT3 activation may be a common signaling mechanism in the pathogenesis of LPS-induced ALI. A study verified that the STAT3 transcription factor is activated in lung injury and promotes macrophage and inflammatory cell infiltration in the lung and BALF, the inhibition of the STAT3 signaling pathway protects the lungs from damage [39]. The RAGE signaling pathway may directly or indirectly lead to the production of pro-inflammatory cytokines, which can also induce endoplasmic reticulum (ER) stress of chronic inflammation, suggesting that RAGE may be a key mediator of ER stress [40]. RAGE activation is even found to prolong and excessive UPR in some disease models [41]. Autophagy is related to ER stress at many levels, autophagy activation occurs under ER stress [42]. However, there is no relevant study on whether RAGE induced autophagy is related to endoplasmic reticulum stress, which is also the direction of our next exploration.

## Conclusions

This research underscores the higher expression of RAGE and autophagic cell death in LPS-treated mouse models and ATII cells. LPS-activated autophagy in ATII cells via a RAGE-dependent signaling pathway, cessation of RAGE exerts protective effects in response to LPS-induced ATII cells injury and inflammation by suppressing autophagy. These findings provide new insights into RAGE mediated autophagy activation as a potential therapeutic strategy for ALI/ARDS.

## Supplementary Information

**Additional file 1: Figure S1**. LPS-induced autophagic death in primary ATII cells depended on RAGE activation. A: Treated primary ATII cells with siRAGE and detected the mRNA expression of RAGE. B: The immunoblots of RAGE, Beclin1, LC3 II/I and cleaved Caspase 3 in primary ATII cells. C: The immunoblot of Beclin1, LC3 II/I and cleaved Caspase 3 in primary ATII cells upon LPS treatment when being pretreated with or without 3-MA. Each experiment was repeated at least three times. D: Immunofluorescence of RAGE in primary ATII cells, spinning disk confocal microscopy at × 40 magnification (scale bar 50 μm). * indicates the significant difference compared with the control group. # indicates the significant difference compared with LPS group. P < 0.05, differences in characteristics between groups were analyzed using the Kruskal–Wallis test with Dunn’s post hoc tests.

**Additional file 2: Figure S2**. The activity of autophagy and survival rate in A549 cells pretreated with or without sRAGE. A: The cell survival rate of A549 cells in response to sRAGE pre-treatment followed by LPS stimulation. Data were obtained from CCK-8 experiments and performed more than three times. B: The immunoblot of RAGE, Beclin1, LC3 II/I and cleaved Caspase 3 in A549 cells upon LPS treatment when being pretreated with or without sRAGE. Each experiment was repeated at least three times. *Indicates the significant difference compared with the control group. #Indicates the significant difference compared with LPS group. P < 0.05, differences in characteristics between groups were analyzed using the Kruskal–Wallis test with Dunn’s post hoc tests.

## Data Availability

The datasets of current study supporting this conclusion are included in this paper.

## Abbreviations

RAGE: Advanced glycation end product receptor
LPS: Lipopolysaccharide
3-MA: 3-Methyladenine
AEC: Alveolar epithelial cell
CCK-8: Cell counting kit-8
ELISA: Enzyme-linked immunosorbent assay
TUNEL: Terminal deoxynucleotidyl transferase dUTP nick-end labelling
BALF: Bronchoalveolar lavage fluid
IL-6: Interleukin-6
IL-8: Interleukin-8
sRAGE: Soluble RAGE
ATI: Type I alveolar epithelial cell
ATII: Type II alveolar epithelial cell
ALI: Acute lung injury
ARDS: Acute respiratory distress syndrome
siRAGE: RAGE small interfering RNA
HE: Hematoxylin and eosin
PBS: Phosphate-buffered saline
PCR: Polymerase chain reaction
mTOR: Mammalian target of rapamycin
UPR: Unfolded protein response

## References

[1] Fan EKY, Fan J (2018). Regulation of alveolar macrophage death in acute lung inflammation. Respir Res.

[2] Ruaro B, Salton F, Braga L, Wade B, Confalonieri P, Volpe MC, Baratella E, Maiocchi S, Confalonieri M. The history and mystery of alveolar epithelial type II cells: focus on their physiologic and pathologic role in lung. Int J Mol Sci 2021, 22(5). 10.3390/ijms22052566 PMC7961977 33806395

[3] Chen H, Bai C, Wang X (2010). The value of the lipopolysaccharide-induced acute lung injury model in respiratory medicine. Expert Rev Respir Med.

[4] Li J, Lu K, Sun F, Tan S, Zhang X, Sheng W, Hao W, Liu M, Lv W, Han W (2021). Panaxydol attenuates ferroptosis against LPS-induced acute lung injury in mice by Keap1-Nrf2/HO-1 pathway. J Transl Med.

[5] Hudson BI, Lippman ME (2018). Targeting RAGE signaling in inflammatory disease. Annu Rev Med.

[6] Uchida T, Shirasawa M, Ware LB, Kojima K, Hata Y, Makita K, Mednick G, Matthay ZA, Matthay MA (2006). Receptor for advanced glycation end-products is a marker of type I cell injury in acute lung injury. Am J Respir Crit Care Med.

[7] Kim HJ, Jeong MS, Jang SB (2021). Molecular characteristics of RAGE and advances in small-molecule inhibitors. Int J Mol Sci.

[8] Li Y, Wu R, Tian Y, Yu M, Tang Y, Cheng H, Tian Z (2015). RAGE/NF-kappaB signaling mediates lipopolysaccharide induced acute lung injury in neonate rat model. Int J Clin Exp Med.

[9] Wang H, Wang T, Yuan Z, Cao Y, Zhou Y, He J, Shen Y, Zeng N, Dai L, Wen F (2018). Role of receptor for advanced glycation end products in regulating lung fluid balance in lipopolysaccharide-induced acute lung injury and infection-related acute respiratory distress syndrome. Shock.

[10] Li J, Wang K, Huang B, Li R, Wang X, Zhang H, Tang H, Chen X (2021). The receptor for advanced glycation end products mediates dysfunction of airway epithelial barrier in a lipopolysaccharides-induced murine acute lung injury model. Int Immunopharmacol.

[11] Jabaudon M, Blondonnet R, Pereira B, Cartin-Ceba R, Lichtenstern C, Mauri T, Determann RM, Drabek T, Hubmayr RD, Gajic O (2018). Plasma sRAGE is independently associated with increased mortality in ARDS: a meta-analysis of individual patient data. Intensive Care Med.

[12] Yamamoto Y, Harashima A, Saito H, Tsuneyama K, Munesue S, Motoyoshi S, Han D, Watanabe T, Asano M, Takasawa S (2011). Septic shock is associated with receptor for advanced glycation end products ligation of LPS. J Immunol.

[13] Izushi Y, Teshigawara K, Liu K, Wang D, Wake H, Takata K, Yoshino T, Takahashi HK, Mori S, Nishibori M (2016). Soluble form of the receptor for advanced glycation end-products attenuates inflammatory pathogenesis in a rat model of lipopolysaccharide-induced lung injury. J Pharmacol Sci.

[14] Oczypok EA, Perkins TN, Oury TD (2017). All the "RAGE" in lung disease: the receptor for advanced glycation endproducts (RAGE) is a major mediator of pulmonary inflammatory responses. Paediatr Respir Rev.

[15] Lukaszewicz A, Cwiklinska M, Zarzecki M, Szoka P, Lachowicz J, Holownia A (2019). Cytotoxicity, oxidative stress, and autophagy in human alveolar epithelial cell line (A549 cells) exposed to standardized urban dust. Adv Exp Med Biol.

[16] Sipos A, Kim KJ, Chow RH, Flodby P, Borok Z, Crandall ED (2018). Alveolar epithelial cell processing of nanoparticles activates autophagy and lysosomal exocytosis. Am J Physiol Lung Cell Mol Physiol.

[17] Hu Y, Lou J, Mao YY, Lai TW, Liu LY, Zhu C, Zhang C, Liu J, Li YY, Zhang F (2016). Activation of MTOR in pulmonary epithelium promotes LPS-induced acute lung injury. Autophagy.

[18] Guo L, Wu X, Zhao S, Zhang X, Qian G, Li S (2021). Autophagy inhibition protects from alveolar barrier dysfunction in LPS-induced ALI mice by targeting alveolar epithelial cells. Respir Physiol Neurobiol.

[19] Blondonnet R, Audard J, Belville C, Clairefond G, Lutz J, Bouvier D, Roszyk L, Gross C, Lavergne M, Fournet M (2017). RAGE inhibition reduces acute lung injury in mice. Sci Rep.

[20] El-Emam SZ (2021). Sesamol alleviates the cytotoxic effect of cyclophosphamide on normal human lung WI-38 cells via suppressing RAGE/NF-kappaB/autophagy signaling. Nat Prod Bioprospect.

[21] Liu B, Zhao H, Wang Y, Zhang H, Ma Y (2020). Astragaloside IV attenuates lipopolysaccharides-induced pulmonary epithelial cell injury through inhibiting autophagy. Pharmacology.

[22] Shao L, Xiong X, Zhang Y, Miao H, Ren Y, Tang X, Song J, Wang C (2020). IL-22 ameliorates LPS-induced acute liver injury by autophagy activation through ATF4-ATG7 signaling. Cell Death Dis.

[23] Liang Y, Yang N, Pan G, Jin B, Wang S, Ji W (2018). Elevated IL-33 promotes expression of MMP2 and MMP9 via activating STAT3 in alveolar macrophages during LPS-induced acute lung injury. Cell Mol Biol Lett.

[24] Guillot L, Nathan N, Tabary O, Thouvenin G, Le Rouzic P, Corvol H, Amselem S, Clement A (2013). Alveolar epithelial cells: master regulators of lung homeostasis. Int J Biochem Cell Biol.

[25] Nova Z, Skovierova H, Calkovska A. Alveolar-capillary membrane-related pulmonary cells as a target in endotoxin-induced acute lung injury. Int J Mol Sci 2019, 20(4). 10.3390/ijms20040831 PMC6412348 30769918

[26] Denton D, Kumar S (2019). Autophagy-dependent cell death. Cell Death Differ.

[27] Zhang X, Zheng J, Yan Y, Ruan Z, Su Y, Wang J, Huang H, Zhang Y, Wang W, Gao J (2019). Angiotensin-converting enzyme 2 regulates autophagy in acute lung injury through AMPK/mTOR signaling. Arch Biochem Biophys.

[28] Ma J, Sun Q, Mi R, Zhang H (2011). Avian influenza A virus H5N1 causes autophagy-mediated cell death through suppression of mTOR signaling. J Genet Genomics.

[29] Liu J, Lv X, Dong W, Hu M, Xu J, Qian G, Li Y (2018). The role of SIRT1 in autophagy in lipopolysaccharide-induced mouse type II alveolar epithelial cells. Inflammation.

[30] Li S, Guo L, Qian P, Zhao Y, Liu A, Ji F, Chen L, Wu X, Qian G (2015). Lipopolysaccharide induces autophagic cell death through the PERK-dependent branch of the unfolded protein response in human alveolar epithelial A549 cells. Cell Physiol Biochem.

[31] Hofmann MA, Drury S, Fu C, Qu W, Taguchi A, Lu Y, Avila C, Kambham N, Bierhaus A, Nawroth P (1999). RAGE mediates a novel proinflammatory axis: a central cell surface receptor for S100/calgranulin polypeptides. Cell.

[32] Piazza O, Leggiero E, De Benedictis G, Pastore L, Salvatore F, Tufano R, De Robertis E (2013). S100B induces the release of pro-inflammatory cytokines in alveolar type I-like cells. Int J Immunopathol Pharmacol.

[33] Brisslert M, Amu S, Pullerits R (2013). Intra-peritoneal sRAGE treatment induces alterations in cellular distribution of CD19(+), CD3 (+) and Mac-1 (+) cells in lymphoid organs and peritoneal cavity. Cell Tissue Res.

[34] Erusalimsky JD (2021). The use of the soluble receptor for advanced glycation-end products (sRAGE) as a potential biomarker of disease risk and adverse outcomes. Redox Biol.

[35] Antonelli A, Di Maggio S, Rejman J, Sanvito F, Rossi A, Catucci A, Gorzanelli A, Bragonzi A, Bianchi ME, Raucci A (2017). The shedding-derived soluble receptor for advanced glycation endproducts sustains inflammation during acute *Pseudomonas aeruginosa* lung infection. Biochim Biophys Acta Gen Subj.

[36] Jabaudon M, Futier E, Roszyk L, Chalus E, Guerin R, Petit A, Mrozek S, Perbet S, Cayot-Constantin S, Chartier C (2011). Soluble form of the receptor for advanced glycation end products is a marker of acute lung injury but not of severe sepsis in critically ill patients. Crit Care Med.

[37] Ramsgaard L, Englert JM, Manni ML, Milutinovic PS, Gefter J, Tobolewski J, Crum L, Coudriet GM, Piganelli J, Zamora R (2011). Lack of the receptor for advanced glycation end-products attenuates *E. coli* pneumonia in mice. PLoS ONE.

[38] Jabaudon M, Blondonnet R, Roszyk L, Pereira B, Guérin R, Perbet S, Cayot S, Bouvier D, Blanchon L, Sapin V (2015). Soluble forms and ligands of the receptor for advanced glycation end-products in patients with acute respiratory distress syndrome: an observational prospective study. PLoS ONE.

[39] Zhao J, Yu H, Liu Y, Gibson SA, Yan Z, Xu X, Gaggar A, Li PK, Li C, Wei S (2016). Protective effect of suppressing STAT3 activity in LPS-induced acute lung injury. Am J Physiol Lung Cell Mol Physiol.

[40] Piperi C, Adamopoulos C, Dalagiorgou G, Diamanti-Kandarakis E, Papavassiliou AG (2012). Crosstalk between advanced glycation and endoplasmic reticulum stress: emerging therapeutic targeting for metabolic diseases. J Clin Endocrinol Metab.

[41] Panda DK, Bai X, Sabbagh Y, Zhang Y, Zaun HC, Karellis A, Koromilas AE, Lipman ML, Karaplis AC (2018). Defective interplay between mTORC1 activity and endoplasmic reticulum stress-unfolded protein response in uremic vascular calcification. Am J Physiol Renal Physiol.

[42] Song S, Tan J, Miao Y, Zhang Q (2018). Crosstalk of ER stress-mediated autophagy and ER-phagy: involvement of UPR and the core autophagy machinery. J Cell Physiol.

